# Functional Role of Non-Coding RNAs during Epithelial-To-Mesenchymal Transition

**DOI:** 10.3390/ncrna4020014

**Published:** 2018-05-28

**Authors:** Almudena Expósito-Villén, Amelia E. Aránega, Diego Franco

**Affiliations:** Cardiovascular Development Group, Department of Experimental Biology, University of Jaén, 23071 Jaén, Spain; aev00006@red.ujaen.es (A.E.-V.); aaranega@ujaen.es (A.E.A.)

**Keywords:** epithelial-to-mesenchymal transition, microRNA, lncRNAs, transcriptional regulation, post-transcriptional regulation

## Abstract

Epithelial-to-mesenchymal transition (EMT) is a key biological process involved in a multitude of developmental and pathological events. It is characterized by the progressive loss of cell-to-cell contacts and actin cytoskeletal rearrangements, leading to filopodia formation and the progressive up-regulation of a mesenchymal gene expression pattern enabling cell migration. Epithelial-to-mesenchymal transition is already observed in early embryonic stages such as gastrulation, when the epiblast undergoes an EMT process and therefore leads to the formation of the third embryonic layer, the mesoderm. Epithelial-to-mesenchymal transition is pivotal in multiple embryonic processes, such as for example during cardiovascular system development, as valve primordia are formed and the cardiac jelly is progressively invaded by endocardium-derived mesenchyme or as the external cardiac cell layer is established, i.e., the epicardium and cells detached migrate into the embryonic myocardial to form the cardiac fibrous skeleton and the coronary vasculature. Strikingly, the most important biological event in which EMT is pivotal is cancer development and metastasis. Over the last years, understanding of the transcriptional regulatory networks involved in EMT has greatly advanced. Several transcriptional factors such as *Snail*, *Slug*, *Twist*, *Zeb1* and *Zeb2* have been reported to play fundamental roles in EMT, leading in most cases to transcriptional repression of cell–cell interacting proteins such as ZO-1 and cadherins and activation of cytoskeletal markers such as vimentin. In recent years, a fundamental role for non-coding RNAs, particularly microRNAs and more recently long non-coding RNAs, has been identified in normal tissue development and homeostasis as well as in several oncogenic processes. In this study, we will provide a state-of-the-art review of the functional roles of non-coding RNAs, particularly microRNAs, in epithelial-to-mesenchymal transition in both developmental and pathological EMT.

## 1. Introduction

Epithelial-to-mesenchymal transition (EMT) is a key biological process involved in a multitude of developmental and pathological events. It is characterized by the progressive loss of cell-to-cell contacts, changes in cell polarity, and cleavage and invasion of the basal lamina produced by actin cytoskeletal rearrangements, leading to filopodia formation and finally to progressive up-regulation of mesenchymal gene expression. At the molecular level, epithelial cells are characterized by the expression of epithelial (E)-cadherin, ZO-1, occludin, cytokeratins, claudins, and type IV collagen, while mesenchymal cells are characterized by vimentin, α-SMA, FSP-1, fibronectin, neural (N)-cadherin, and secretion of type I and III collagens. Transition from an epithelial to mesenchymal phenotype is produced by up-regulation of a subset of transcription factors, such as *Snail/Slug*, *Twist* and *Zeb1/2*. Regulatory mechanisms driving the expression of these transcription factors is modulated by several signaling pathways, including TGF-β/BMP, Notch, Wnt/β-catenin, receptor tyrosine kinase (Rtk) and Hedgehog [1,2]. Therefore, regulation of EMT is a complex process that is tightly regulated in different biological events. Epithelial-to-mesenchymal transition is a reversible process, and therefore in certain circumstances mesenchymal-to-epithelial transition (MET) can also take place, particularly during oncogenic processes [3,4,5]. Regulatory mechanisms driving MET fall beyond the scope of the present review (see for excellent reviews on MET [6,7,8]).

First EMT events during embryogenesis are observed at very early developmental stages, i.e., during gastrulation. Epiblast cell layer receives positional information to initiate an antero-posterior differentiation from epithelial cells to the underlying space leading to the formation of the third embryonic layer, the mesoderm. Epithelial-to-mesenchymal transition is a widely conserved developmental process during gastrulation across the animal kingdom, spanning from cnidarians [9,10], insects [11], fishes [12], and avians [13,14] to mammalians [15,16,17], using a wide variety of different signaling pathways [18,19,20,21]. Additional EMT events occur during embryonic development of several organs, such as gonadal development [22], the formation of the eye lens and retinal pigmented epithelium [23,24,25,26,27,28,29,30,31], the tubulogenesis and renal epithelial morphogenesis during kidney development [32,33,34], the formation of the stromal cells in the liver [35], the formation of β-pancreatic cells [36,37], and also during cardiovascular system development [38,39,40], involving signaling pathways such as TGF-β/BMP [41,42,43] and Notch [44]. In fact, during cardiac development, two separate EMT events take place, one during the formation of the atrioventricular and semilunar valves [45,46,47], a process that is highly conserved in fishes [48,49] and avian and mammalian species [50,51,52]. In addition, a second EMT process occurs during cardiac development after the initial epicardial layer formation and subsequent invasion into the developing ventricular chambers, involving similar upstream signaling pathways, i.e., TGF-β/BMP signaling [53,54,55,56,57], but curiously independently of *Snail*, at least in mice [58], and thus requiring additional regulatory player such as PDGF [59], Wt1 and Tbx18 [60,61].

In addition to those developmental events in which EMT plays a fundamental role, EMT is also determinant in several pathophysiological conditions, in particular in cancer. Multiple sets of evidence demonstrate that EMT is involved in oncogenic processes of different tissues such as in the gonads in the development of ovarian surface epithelial cancer [62,63,64,65], in the kidney in clear cell renal cell carcinoma [66,67,68,69,70], in the lungs leading to both adenocarcinoma and non-small cellular lung cancer (NSCLC) [71,72,73,74,75], in the liver in cholangiocarcinoma and hepatocellular carcinoma [76,77,78,79], and in the pancreas [80,81,82,83] as well as in other tissues [84,85,86,87,88,89,90,91].

Over the last decade our understanding of the molecular mechanisms driving EMT has greatly advanced. Multiple triggering signals, including TGF-β/BMP, Notch, Wnt/β-catenin, Rtk and Hedgehog signaling pathways converge into the activation of a core set of transcription factors [1,2] that initiate the conversion of epithelial to mesenchymal cells. In addition to the transcriptional modulation, a novel layer of gene regulatory mechanism is emerging, i.e., non-coding RNAs. Non-coding RNAs constitute a wide variety of RNA molecules with different functional and structural characteristics, including, among others, microRNAs (miRNAs) and long non-coding RNAs (lncRNAs) [92,93,94]. The implication of both types of non-coding RNAs in EMT has been revealed in embryonic development [95,96,97,98], cellular homeostasis [99,100,101], and physiopathological conditions [102,103,104], and more extensively in oncogenic diseases, such as for example those affecting the urogenital system [105,106,107,108,109,110,111,112,113]. Importantly, cross-talk between both types of non-coding RNAs (ncRNAs) is also emerging as demonstrated in multiple oncogenic settings, including renal cell carcinoma [114], ovarian cancer [115], squamous cell carcinoma [116], and hepatocellular carcinoma [117,118]. Importantly, ncRNAs are also equally implicated in the reverse mode of EMT, i.e., MET [119,120]. In the following subheadings the functional contribution of microRNAs in particular, and to a lesser extent long non-coding RNA, will be discussed and their interconnections on the regulation of core transcriptional regulatory mechanisms driving EMT in development and disease. The emerging role of lncRNAs in EMT have been recently revised in different pathological conditions [118,121]. Some evidence demonstrates that microRNAs can regulate EMT by modulating upstream signaling pathways such as TGF-β [122,123,124,125,126,127,128,129,130,131], Wnt/β-catenin [132,133,134], Notch [135], and Hedgehog [136] signaling, respectively, both in normal and pathological conditions. We have focused this review on dissecting the functional roles of microRNAs and lncRNAs within two levels of EMT processes. Firstly, the regulatory roles of these ncRNAs in the expression and function of those transcription factors involved in the initiation of the EMT process are elucidated, and secondly, a report on the regulation of those proteins that effectively confer migratory capacities to those mesenchymal cells is provided.

## 2. Contribution of microRNAs to Epithelial-To-Mesenchymal Transition Regulation

MicroRNAs are a subset of small non-coding RNAs of 22–24 nucleotides in length that can post-transcriptionally modulate gene expression by binding to the 3′ untranslated region of coding RNAs by base-pair complementarity and inducing protein translation blockage and/or messenger RNA (mRNA) degradation [92]. Such capabilities are provided by the seed sequence, i.e., six nucleotides stretch spanning from positions 2 to 8 in the mature microRNA molecule. MicroRNAs are encoded in the nucleus, transcribed by RNA polymerase II and polyadenylated in form of pre-miRNAs and/or pri-miRNA precursor molecules depending whether they are derived from monocystronic or polycistronic miRNAs molecules, respectively [92]. At present over 1500 microRNAs have been identified in humans (http://www.mirbase.org). MicroRNAs that share the same functional seed sequence, such as for example miR-195a and miR-195b, even if they are located at different chromosomal positions, exert similar functional capabilities.

At present at least five different transcription factors have been implicated directing EMT in both normal and pathological conditions; i.e., *Snail*, *Slug*, *Twist*, *Zeb1* and *Zeb2*. Our current understanding on the regulation of these transcription factors by microRNAs during embryonic development and/or during tissue homeostasis is scarce. Several sets of evidence demonstrate that a limited number of microRNAs can regulate *Twist* [137] and/or *Zeb1/Zeb2* [138] during development and/or tissue homeostasis. In the cardiovascular system our understanding of the regulatory roles of microRNAs during EMT is also in its infancy. Three microRNAs have been involved in EMT during endocardial cushion formation [139,140,141], i.e., miR-23, miR-126 and miR-199, all of them inhibiting EMT progression. Their functional properties are conserved in mice, chicken and zebrafish, supporting a wide evolutionary conservation. During epicardial to mesenchymal transition, our understanding of the regulatory role of microRNAs is also rather scarce [96,97,98]. Deletion of the microRNA processing enzyme *Dicer* leads to impaired epicardial formation, yet EMT is not severely affected [98]. In this context, miR-21 manipulation leads to impaired EMT progression and fibrogenic cell differentiation of epicardial-derived cells. On the other hand, in pathological conditions, all these transcription factors are distinctly modulated by microRNAs.

### 2.1. Regulation of Snail by microRNAs

**Figure 1:**
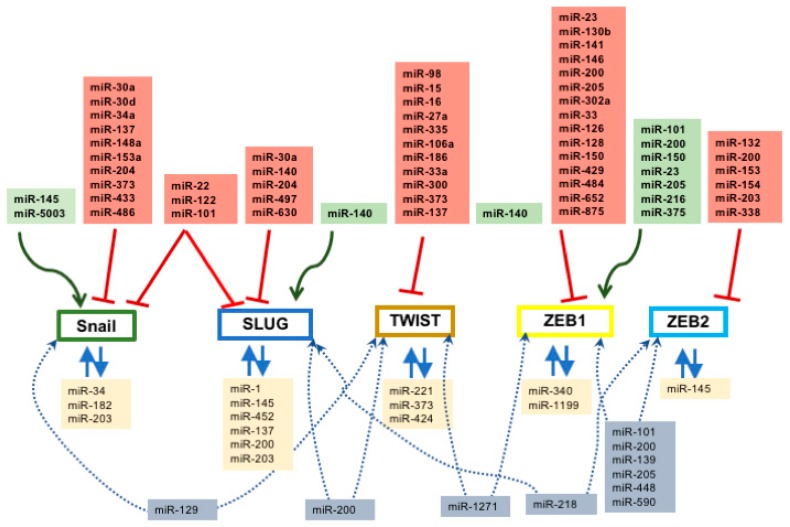
Schematic representation of the microRNAs regulating the epithelial-to-mesenchymal transition (EMT)-associated transcription factors *Snail*, *Slug*, *Twist*, *Zeb1*, and *Seb2*. Red lines demarcate inhibitory pathways, green lines demarcate activation pathways. Dotted lines delineate those microRNAs modulating the expression of different transcription factors, whereas blue arrows display regulatory loops.

A bulk of microRNAs have been identified to down-regulate *Snail* expression, blocking therefore the EMT process (Figure 1). miR-30a directly targets *Snail*, and therefore blocks EMT progression in NSCLC and serves as a potential biomarker for this oncogenic entity [142]. miR-30d, miR-137 and miR-34a block EMT and cell invasion in ovarian cancer [143,144] while miR-148a and miR-153a exert similar functions via *Snail* down-regulation in hepatocellular carcinoma [145,146]. miR-204 and miR-486 directly block *Snail* expression in gastric cancer [147] and prostate cancer [148], respectively, suppressing EMT, invasion, migration, and metastasis. Importantly, miR-148a indirectly regulate *Met* which in turn leads to *Snail* downregulation [145] in hepatocellular carcinoma, and similar indirect effects have also been reported for miR-374 targeting *Foxc1* in cervical cancer [149] and miR-433 targeting *Met/Creb1* in bladder cancer [150], leading to *Snail* inhibition. Overall, these observations demonstrate that multiple microRNAs can target *Snail*, directly or indirectly, having similar phenotypical consequences.

Several microRNAs can modulate *Snail* up-regulation therefore promoting EMT and thus cell migration, metastasis, and invasion (Figure 1). In this context, miR-145 down-regulation leads to enhanced *Snail* expression and increased EMT in osteosarcoma [151] and miR-5003 in breast cancer [152].

### 2.2. Regulation of Slug by microRNAs

In addition to *Snail*, EMT is also controlled by *Slug* and therefore a subset of microRNAs has also been described to inhibit *Slug* expression and thus EMT progression (Figure 1). miR-30a and miR-497 inhibit EMT and metastasis in breast cancer [153,154] while miR-204 and miR-630 inhibit EMT and tumor metastasis by targeting *Slug* in cholangiocarcinoma [155] and hepatocellular carcinoma [156], and miR-203 targets *Slug* in glioblastoma, promoting EMT [157]. On the other hand, downregulation of miR-140 leads to up-regulation of *Slug* and therefore to increased invasion in esophageal cancer [158].

### 2.3. Co-Regulation of Snail and Slug by microRNAs

Importantly, several microRNAs can directly influence expression of both *Snail* and *Slug*, promoting a more robust modulation of the EMT process. In the urogenital system, miR-22 plays a role controlling *Snail/Slug* expression in bladder cancer [159]. In the digestive tract, miR-122 is pivotal in hepatocellular carcinoma (HCC) [133] and miR-101 in oral tongue squamous cell carcinoma [160] (Figure 1).

Furthermore, auto-regulatory loops between different microRNAs and *Snail* and *Slug* have been described. In particular, *Snail* can regulate expression of miR-3 and this microRNA can also regulate *Snail* expression [144,161,162], a regulatory mechanism that has indeed been described in both normal tissue homeostasis [161,162] and pathologic contexts such as ovarian cancer [162]. miR-34 directly targets *Snail* 3′ untranslated region (UTR), leading to *Snail* down-regulation, while *Snail*, in conjunction with other EMT-related transcription factors such as *Zeb1*, binds the miR-34 promoter generating such a regulatory loop. In addition, *Slug* can mediate miR-452, miR-137, and miR-145 expression by directly binding to their promoters, leading in this way to tumor invasion and metastasis [163,164,165]. miR-203 can promote a feedback regulatory loop with *Snail* interacting at the same time with the miR-200–Zeb1 regulatory loop [166]. Similar regulatory loops are also reported for miR-182–*Snail* blocking metastasis in breast cancer [167], miR-1–*Slug* and miR-200–*Slug* inhibiting tumorigenesis in prostate cancer [168], and miR-203–*Slug* in breast cancer [169].

### 2.4. Regulation of Twist by microRNAs

The transcription factor *Twist* is also modulated by multiple microRNAs during EMT in several tissues (Figure 1). miR-98 regulates *Twist* expression in the respiratory system, in particular in NSCLC [170]. In the digestive tract, several microRNAs play a role in distinct oncogenic processes, miR-15, miR-16, miR-335 and miR-137 are involved in gastric cancer [171,172] and colorectal cancer [173], while miR-27a is involved in hepatic cancer [174] and miR-106a in endometrial cancer [175] leading in both cases to direct downregulation of *Twist* by targeting its 3′ UTR. miR-186 also regulate *Twist* expression in the oncogenic processes of the urogenital tract, in particular in ovarian [176] and prostate cancer [177] while miR-300 can inhibit *Twist* expression leading to EMT blockage in melanoma [178]. Importantly, down-regulation of miR-106a, mediated by PDGF signaling, leads to *Twist* up-regulation and thus EMT progression in hepatoma cells [179] while the opposite phenotypical consequences are observed by the IL6-mediated regulation of miR-33a leading to *Twist* downregulation and thus suppression of tumor progression in gallbladder cancer [180]. Feed-forward mechanisms also provide regulatory loops for miR-373, miR-221, miR-424, and *Twist* [181,182] in different oncogenic processes, involving targeting of the *Twist* 3′UTR by the corresponding miRNAs and promoter regulation by *Twist*. In addition, miR-221 and miR-424, Twist-regulated microRNAs, are also involved in EMT regulation, leading to increased metastasis in cervical cancer [183] and breast cancer [184].

### 2.5. Regulation of Zeb1 and Zeb2 by microRNAs

The largest number of microRNAs are known to modulate *Zeb1* and *Zeb2* expression during EMT (Figure 1). In particular, several of these microRNA–*Zeb1/Zeb2* interactions have been reported in normal and pathological adult tissues. miR-130b [185], miR-146 [186,187], miR-200 [188,189,190,191,192], and miR-205 [193] regulate *Zeb1*, modulating EMT under normal physiological conditions, while miR-302a regulates EMT in diabetic kidney disease [194]. Importantly, miR-200 family members [192,195] can also modulate both *Zeb1* and *Zeb2* simultaneously and evidence shows that *Snail* can be equally modulated [196]. In oncogenic conditions, a large array of microRNAs have also been reported to inhibit *Zeb1* expression leading therefore to EMT blockage: miR-33 in adenocarcinoma [197], miR-126 in osteosarcoma [198], miR-128 in esophageal squamous cell cancer [199], miR-150 [200] and miR-200 in ovarian [201] and breast cancer [202], miR-205 [203] and miR-429 [204] and miR-484 [205] in cervical carcinoma, miR-652 in pancreatic cancer [206], miR-875 in prostate cancer [207], miR-23 in bladder cancer [208] and miR-205 in glioblastoma [209]. On the other hand, up-regulation of *Zeb1* is also modulated by several microRNAs such as miR-101 [210], miR-200 and miR-150 in colon carcinoma [211,212] and indirectly by miR-200 downregulation in breast cancer [213]. Furthermore, *Zeb1* can directly regulate miR-375 expression by directly binding to its promoter, leading to transcriptional repression and thus activation of EMT in prostate cancer [214].

*Zeb2* is also regulated by a large number of microRNAs. Several of them inhibit *Zeb2* expression leading to decreased EMT, such as miR-132 in colorectal cancer [215] and lung cancer [216], miR-153 in ovarian cancer [217], miR-154 in NLCLC [218], miR-203 in adenocarcinoma [219] and nasopharyngeal carcinoma [220], miR-338 in gastric cancer [221], and members of the miR-200 family in glioma cells [222], gastric carcinoma [223,224], and NSCLC [225], while no evidence has been reported to date on up-regulation of *Zeb2* by microRNA deregulation.

Similar to *Snail/Slug* and *Twist*, feed-forward regulatory mechanisms are operative for *Zeb1/Zeb2* and microRNAs. Reciprocal repression between the *Zeb1* and miR-200 family promotes EMT and thus cancer migration and invasion [226,227], a regulatory mechanism that is also reported for miR-1199–*Zeb1* interactions [228]. Likewise, miR-340 and *Zeb1* display a feed-forward loop involved in breast cancer progression [229], and miR-145 and *Zeb2* leading to prostate cancer [230].

Furthermore, a large set of microRNAs co-regulates expression of both *Zeb1* and *Zeb2*, modulating therefore tumor progression, for example miR-101 in ovarian carcinoma [231], miR-200 [232,233,234,235] in gastric adenocarcinoma, miR-139 in HCC [236], miR-205 [237] and miR-448 in breast cancer [238], and miR-590 in glioblastoma [239].

### 2.6. Co-Regulation of Epithelial-To-Mesenchymal Transition-Associated Transcription Factors by microRNAs

Importantly, co-regulation between these transcription factors is also applicable. miR-129 can simultaneously regulate *Twist* and *Snail* expression [182], miR-200 co-regulates *Slug* and *Zeb1*, miR-1271 co-regulates *Twist* and *Zeb1* [240], miR-218 regulates *Slug* and *Zeb2* [241] and miR-200 co-regulates *Zeb1* and *Snail* [196,242]. These data demonstrate a complex and diverse regulatory role of microRNAs in EMT progression.

### 2.7. Regulation of Cell–Cell Contact and Cytoskeletal Proteins by microRNAs

**Figure 2:**
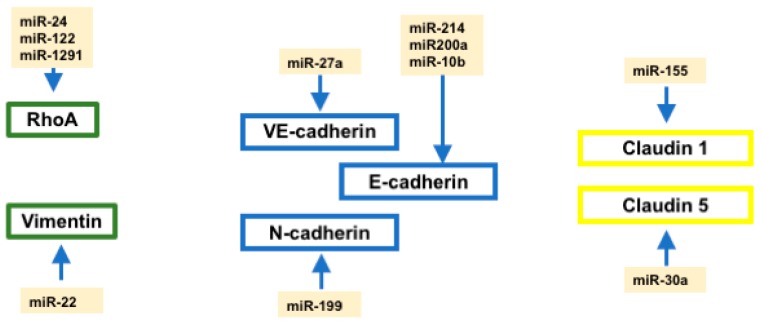
Schematic representation of the microRNAs regulating cytoskeletal and cell–cell contact proteins associated with EMT progression. Abbreviations: VE-cadherin, vascular-endothelial cadherin; E-cadherin, epithelial cadherin; N-cadherin, neural cadherin.

In addition to the regulatory mechanisms exerted by microRNAs at the level of EMT-associated signaling pathways and key orchestrating transcription factors, several studies also pointed out a role for microRNAs directly regulating cell–cell contact and cytoskeletal proteins (Figure 2). Three different microRNAs—miR-122, miR-24, and miR-1291—can directly target RhoA expression [124,243,244] while miR-22 can similarly influence vimentin expression [158] providing therefore signaling cues to modify the cytoskeletal organization during the transition from epithelium to mesenchyme. A number of microRNAs have been reported to regulate cadherin and claudin expression (Figure 2). In particular, miR-10b and miR-214 have been reported to directly regulate E-cadherin by targeting its 3′UTR [105,245], while indirect regulation of E-cadherin is mediated by multiple microRNAs such as e.g., members of the miR-200 family cadherin [188,232,233,234]. Furthermore, miR-27a directly regulates vascular endothelial (VE)-cadherin [173], while miR-199a modulates N-cadherin expression [126]. Claudin 1 and Claudin 5 are also directly targeted by microRNAs, such as miR-155 and miR-30a, respectively [246,247]. Importantly, evidence on the cytoskeletal and cell–cell contact proteins has been only reported in oncogenic processes, while no reports are currently available for normal embryological and/or homeostatic processes.

In addition to those mechanisms driving microRNA-mediated regulation of EMT transcription factors, cell–cell contact, and cytoskeletal proteins, a large number of additional studies have reported the fundamental role of microRNAs regulating additional molecular targets involved in EMT, in particular during oncogenic processes [248,249,250,251,252,253,254,255,256,257]. Among these mechanisms it is important to highlight the emerging functional role of epigenetic regulation of microRNAs during EMT. Several studies reported that hypermethylation and/or demethylation of miRNA promoters can influence expression of EMT progression, such as for miR-211 [258,259] in melanoma cells, miR-129 [260] in HCC, miR-124 [261] in endometrial cancer, and miR-200 [262] and miR-203 [263] in distinct cell types. In some cases, epigenetic modulation of miRNA expression leads to up-regulation of TGF-β signaling such as for miR-142 [264] in HCC or to epigenetic modulators such as HDAC5 [265] in NSCLC. Alternatively, miRNAs can directly target epigenetic modulators such as DNMT1 by miR-152 [266] or lysine K demethylase 6B by miR-941 [267] leading to impaired EMT development, respectively. Importantly, the epigenetic regulation of EMT involves multiple microRNAs within different tissues. For further reading, excellent reviews have been recently published on the topic [268,269,270,271,272,273]. There is also emerging evidence on the role of epigenetics in MET [274]. Additional studies will be therefore required to fully implement the cross roads between epigenetics microRNAs, EMT/MET and all these molecular partners.

## 3. Contribution of Long Non-Coding RNAs to Epithelial-To-Mesenchymal Transition Regulation

In contrast to microRNAs, our current understanding of long non-coding RNAs is limited. The development of new massive sequencing techniques has led to the discovery and annotation of a large number of long non-coding RNAs, i.e., 96,308 lncRNA genes [275]. These estimates indicate that the number of lncRNAs is twice that of coding genes, supporting an important role of these lncRNA transcripts in multiple biological contexts. Recently, lncRNAs have emerged as major players in regulating gene expression, both at transcriptional and post-transcriptional levels, and they have been implicated in development, stem cell differentiation, cellular homeostasis, and disease [276,277,278]. Long non-coding RNAs display essentially no potential to code for proteins, although structurally are similar to mRNAs. They are normally transcribed by RNA polymerase II, have typical histone modifications, have 5′ terminal cap and 3′ terminal poly(A) tails, are structured by exons and introns, and are often spliced. Although the vast majority of lncRNAs are located in the nuclear genome, lncRNAs are also reported within the mitochondrial DNA. Mitochondrial-encoded lncRNAs are transcribed and processed by mitochondrial transcriptional machinery but are regulated by nuclear encoded proteins [278,279]. Importantly, although lncRNAs are referred to as non-coding, some lncRNAs contain short open reading frames (ORFs) and can be engaged by ribosomes, and thus can generate oligopeptides, although this is mostly limited to sporadic cases.

**Figure 3:**
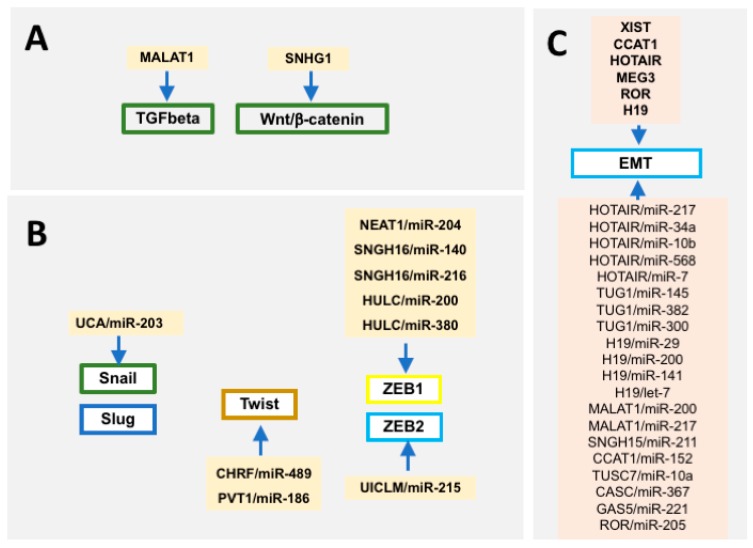
Schematic representation of the long non-coding RNA (lncRNAs) regulating the EMT-associated transcription factors *Snail*, *Slug*, *Twist*, *Zeb1*, and *Zeb2*.

Over the last years the relevance of long non-coding RNAs has emerged. Multiple studies demonstrate that differential expressions of lncRNAs can serve as biomarkers in different oncogenic conditions [280,281,282,283]. A large array of evidence demonstrates that distinct lncRNAs can influence EMT [284,285,286,287,288,289,290,291,292] by regulating upstream signaling pathways such as TGF-β [293,294] or Wnt/β-catenin [295] (Figure 3). In addition, lncRNAs such as UCA1 can regulate *Slug* expression [296], PVT1 and CHRF influence *Twist* function [297,298], and several other lncRNAs such as SNHG16, NEAT1 and HULC have been reported to modulate *Zeb1* [299,300,301,302] and UICLM to *Zeb2* [303] function, respectively (Figure 3).

## 4. Cross-Talk between microRNAs and Long Non-Coding RNAs in Epithelial-To-Mesenchymal Transition

Functional evidence has demonstrated that microRNAs can target lncRNAs, influencing therefore the functional role of these non-coding RNA molecules, and similarly, microRNA expression and function can be modulated by lncRNAs by acting as competing endogenous RNAs (ceRNAs) or as microRNA sponges. Evidence demonstrates that such microRNA-lncRNA cross-talks are operative in oncogenic processes (Figure 3). For example, TUG1 and miR-145 establish a double-negative feedback loop promoting EMT in human bladder cancer cells [304], while H19 and miR-141 regulate cell proliferation and migration in gastric cancer [305]. In fact, a large array of lncRNAs can act as ceRNAs or sponges of EMT-related microRNAs unraveling novel molecular pathways that promote and/or inhibit EMT [306,307,308,309,310,311,312,313,314,315]. For example, lncRNA SNHG15 sponges miR-211 in breast cancer [306] and CCAT1 sponges miR152 in cholangiocarcinoma [307]. Importantly, a single lncRNA can sponge several microRNAs in different cellular contexts—H19 for example can modulate miR-29 and miR-200 expression in bladder [309] and breast [120] cancer, respectively. On the other hand, a single microRNA can be sponged by several lncRNAs, for example miR-200 by H19 [120] and MALAT1 [310] in separate biological contexts, i.e., breast and endometrium cancer, respectively. Furthermore, within the same oncogenic process, distinct lncRNA–microRNA interactions have been described, as in hepatocellular carcinoma leading to Unigene56159–miR-140 [311], TUSC7–miR-109 [312], and CASC2–miR-367 [314] interactions, respectively. In other contexts, the regulatory roles of lncRNAs are manifested by modulation of microRNA expression, yet the precise molecular regulatory circuitries remain to be elucidated [315,316,317,318,319,320,321,322,323,324,325].

## 5. Conclusions and Perspectives

**Figure 4:**
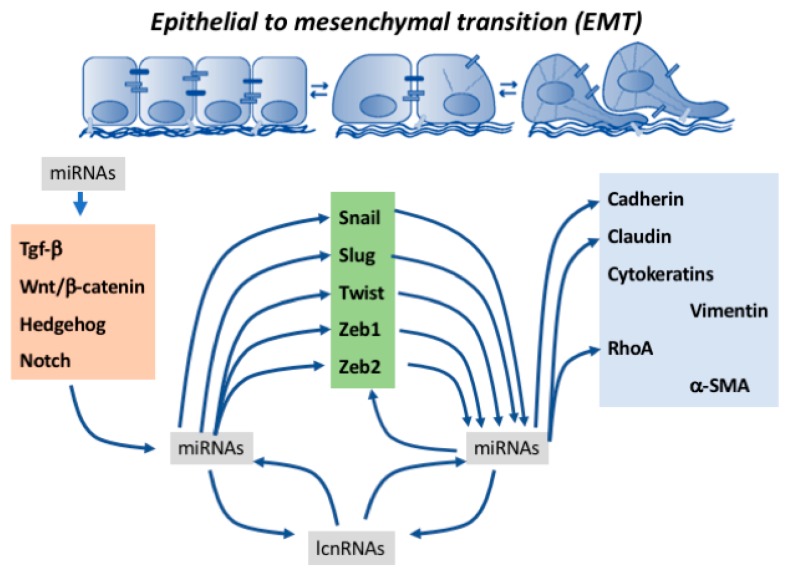
Schematic representation of the microRNA–lncRNA interactions regulating EMT progression.

In this review we have highlighted the pivotal role of non-coding RNAs in several aspects of the epithelial-to-mesenchymal transition. Evidence demonstrates that EMT activators, i.e., upstream signaling pathways such as TGF-β and Wnt signaling, can modulate the expression of a wide variety of microRNAs impacting on the functional properties of the EMT regulators (i.e., transcription factors) and thus on EMT progression. Similarly, transcription factor regulators of EMT can control the expression of a variety of microRNAs having an impact on individual EMT effectors, leading to cell–cell contact and cytoskeletal rearrangements. Thus, these data demonstrate a complex interplay between EMT activators, regulators, and effectors, and microRNAs. Furthermore, a novel layer of complexity is added as an additional subtype of non-coding RNAs, i.e., long non-coding RNAs can also interact with microRNAs, unraveling novel gene regulatory networks in the progression and development of EMT (Figure 4).

Our understanding on the molecular mechanisms of EMT and the implication of the signaling pathways involved, as well as transcription factors driving EMT during embryonic development, has greatly advanced over the last decade. Surprisingly, our current understanding of the regulatory roles exerted by non-coding RNAs during embryonic development is scarce, as it is during adult tissue homeostasis. On the other hand, a large set of evidence has been reported in oncogenic processes, providing evidence that non-coding RNAs such as microRNAs and lncRNAs influence cell tissue homeostasis favoring in many cases pathological EMT. Additionally, they can also serve as potential biomarkers of ongoing oncogenic diseases and most importantly will potentially be used as therapeutic tools.

Mechanistically, multiple microRNAs have been described to target EMT-associated transcription factors such as *Snail*, *Slug*, *Twist*, *Zeb1*, and *Zeb2*, respectively, in different cellular substrates. It is well-known that a single mRNA transcript can be targeted by multiple microRNAs and similarly that a single microRNA can target multiple mRNA transcripts. Thus, it is not surprising that multiple microRNAs can target these EMT transcription factors. However, with the exception of the miR-200 family, current evidence mostly demonstrates a tissue-specific role for a single microRNA, highlighting the importance of the tissue-specific expression of these non-coding RNAs and their corresponding deregulation in a tissue-specific manner. To date, information on the cellular distribution of these regulatory molecules remains scarce.

In addition to microRNAs, a novel layer of complexity on the regulation of EMT progression is emerging as long non-coding RNAs have been widely demonstrated to play pivotal roles. Several sets of evidence demonstrate that lncRNAs can act as sponge of distinct microRNAs, therefore impacting on the progression of EMT. In other cases, lncRNAs can regulate microRNAs by, to date, unclear mechanisms. Given the wide variety of functional properties revealed for lncRNAs in other biological contexts, it is plausible that in the near future, additional roles for these non-coding RNAs will be identified in the context of EMT development. In this setting, lncRNAs can also be postulated as biomarkers of oncogenic diseases, and manipulation of these emerging regulatory molecules might also serve in the near future as a therapeutic tool to control EMT progression and the fatal consequences of pathological EMT, i.e., migration, invasion, and metastasis.
